# PET/MR Imaging of Somatostatin Receptor Expression and Tumor Vascularity in Meningioma: Implications for Pathophysiology and Tumor Outcomes

**DOI:** 10.3389/fonc.2021.820287

**Published:** 2022-01-28

**Authors:** Michelle Roytman, Sean Kim, Shannon Glynn, Charlene Thomas, Eaton Lin, Whitney Feltus, Rajiv S. Magge, Benjamin Liechty, Theodore H. Schwartz, Rohan Ramakrishna, Nicolas A. Karakatsanis, Susan C. Pannullo, Joseph R. Osborne, Jonathan P. S. Knisely, Jana Ivanidze

**Affiliations:** ^1^Departments of Radiology, Weill Cornell Medicine/NewYork-Presbyterian Hospital, New York, NY, United States; ^2^Weill Cornell Medical College, Weill Cornell Medicine/NewYork-Presbyterian Hospital, New York, NY, United States; ^3^Departments of Radiology, New York-Presbyterian Hospital/Columbia University Medical Center, New York, NY, United States; ^4^Department of Neurology, Weill Cornell Medicine/NewYork-Presbyterian Hospital, New York, NY, United States; ^5^Department of Pathology and Laboratory Medicine, Weill Cornell Medicine/NewYork-Presbyterian Hospital, New York, NY, United States; ^6^Department of Neurological Surgery, Weill Cornell Medicine/NewYork-Presbyterian Hospital, New York, NY, United States; ^7^Department of Radiation Oncology, Weill Cornell Medicine/NewYork-Presbyterian Hospital, New York, NY, United States

**Keywords:** meningioma, somatostatin receptor, DOTATATE, PET/MRI, DCE = dynamic contrast enhanced, DCE Perfusion MRI

## Abstract

**Background and Purpose:**

Meningiomas, the most common primary intracranial tumor, are vascular neoplasms that express somatostatin receptor-2 (SSTR2). The purpose of this investigation was to evaluate if a relationship exists between tumor vascularity and SSTR2 expression, which may play a role in meningioma prognostication and clinical management.

**Materials and Methods:**

Gallium-68-DOTATATE PET/MRI with dynamic contrast-enhanced (DCE) perfusion was prospectively performed. Clinical and demographic patient characteristics were recorded. Tumor volumes were segmented and superimposed onto parametric DCE maps including flux rate constant (*Kep*), transfer constant (*Ktrans*), extravascular volume fraction (*Ve*), and plasma volume fraction (*Vp*). Meningioma PET standardized uptake value (SUV) and SUV ratio to superior sagittal sinus (SUVR_SSS_) were recorded. Pearson correlation analyses were performed. In a random subset, analysis was repeated by a second investigator, and intraclass correlation coefficients (ICCs) were determined.

**Results:**

Thirty-six patients with 60 meningiomas (20 WHO-1, 27 WHO-2, and 13 WHO-3) were included. Mean *Kep* demonstrated a strong significant positive correlation with SUV (r = 0.84, p < 0.0001) and SUVR_SSS_ (r = 0.81, p < 0.0001). When stratifying by WHO grade, this correlation persisted in WHO-2 (r = 0.91, p < 0.0001) and WHO-3 (r = 0.92, p = 0.0029) but not WHO-1 (r = 0.26, p = 0.4, SUVR_SSS_). ICC was excellent (0.97–0.99).

**Conclusion:**

DOTATATE PET/MRI demonstrated a strong significant correlation between tumor vascularity and SSTR2 expression in WHO-2 and WHO-3, but not WHO-1 meningiomas, suggesting biological differences in the relationship between tumor vascularity and SSTR2 expression in higher-grade meningiomas, the predictive value of which will be tested in future work.

## Introduction

Meningiomas, highly vascular neoplasms arising from the arachnoid cap cells, are the most common primary intracranial tumor, accounting for approximately 40% of all primary brain tumors (1). Various histopathological classification systems have been utilized for their characterization, with the current 2021 World Health Organization (WHO) classification of central nervous system (CNS) tumors recognizing 15 distinct histological subtypes, subdivided into three grades—Grade 1 (benign), Grade 2 (atypical), and Grade 3 (malignant)—relying largely on mitotic rate, histological, and cytomorphological criteria (2, 3). Higher grade meningiomas have been reported to exhibit increased vascularity, likely related to higher levels of vascular endothelial growth factor (VEGF) and microvessel density, which are surrogate markers for angiogenesis (4). However, meningioma vascularity remains a complex topic. While approximately 80% of meningiomas are considered benign and managed with observation or curative surgical resection, a subset of meningiomas demonstrate aggressive features and may recur or progress despite intervention (5). Importantly, histopathological WHO criteria has been found to be a poor predictor of clinical course, as a subset of patients with grade 2 meningiomas demonstrate a benign course while up to 20% of patients with grade 1 meningiomas experience recurrence (2, 6). Upon failing surgical and/or radiotherapeutic treatments, prognosis is often poor with no available effective medical treatment options, despite numerous clinical trials investigating the use of medications such as temozolomide, hydroxyurea, irinotecan, imatinib, erlotinib, gefitinib, bevacizumab, sunitinib, everolimus, and trabectidin (2).

Surgical resection is the mainstay of treatment for most meningiomas, with postresection Simpson grade serving as a strong predictor of outcome (2). Patients with WHO Grade 1 meningiomas typically undergo imaging and clinical observation, while patients with WHO Grade 3 meningiomas often undergo postsurgical radiation (RT). However, the use of RT post-initial resection in WHO Grade 2 meningiomas is controversial and varies in clinical practice across institutions, with an overall consensus that RT improves outcomes. Advanced adjunct imaging modalities are emerging as potential tools for the management of WHO Grade 2 meningiomas, most notably the use of [^68^Ga]-DOTATATE, a positron emission tomography (PET) radiotracer targeting somatostatin receptor 2 (SSTR2), which has been immunohistochemically proven to be present on the cell surface of 79%–100% of meningiomas (7, 8), and other Gallium-68-labeled somatostatin analogs (9, 10). [^68^Ga]-DOTATATE binds to SSTR2 on the cell surface of meningiomas, with high specificity, serving as an imaging biomarker for the detection of meningiomas. While vascularity may aid in transporting the radiotracer to its destination, vascularity in itself would not explain the sustained binding identified in meningiomas. Additionally, while higher grade meningiomas have been reported to exhibit increased vascularity, such a correlation does not exist between WHO grade and degree of [^68^Ga]-DOTATATE avidity. [^68^Ga]-DOTATATE PET has demonstrated promise in the assessment of resected/irradiated meningiomas and in the assessment of treatment-naive meningiomas by allowing for improved diagnosis and evaluation of extent of disease (11). In addition to serving as a potential predictive imaging biomarker, SSTR2 may serve as a potential therapeutic target utilizing peptide receptor radionuclide therapy (PPRT) *via* ^177^Lutetium[^177^Lu]-DOTATATE. [^177^Lu]-DOTATATE is currently being investigated in two prospective clinical studies (NCT03971461 and NCT04082520) for patients with progressive intracranial meningiomas, serving as a potential novel therapeutic option in the arena of precision medicine (2, 11).

Dynamic contrast-enhanced MRI (DCE-MRI) is an advanced imaging modality allowing for *in vivo* evaluation of tissue perfusion and blood–brain barrier (BBB) disruption (12, 13). Perfusion imaging, which may be performed as DCE-MRI or as dynamic susceptibility contrast (DSC-MRI), is increasingly utilized in clinical practice for both primary and secondary brain neoplasms. In the context of meningiomas, perfusion MRI has been shown to successfully distinguish between lower and higher grades, guide in the differentiation between meningiomas and dural-based metastases, provide information regarding RT response, and provide useful information regarding peritumoral edema surrounding meningiomas, indicative of BBB disruption (13–17).

While meningiomas have distinct and often pathognomonic conventional imaging features (e.g., dural-based, extra-axial, homogeneous enhancement, dural tail, associated hyperostosis), conventional gadolinium-enhanced MRI has its limitations, including the inability to reliably distinguish between meningioma subtypes/grades and challenges discerning posttreatment change from residual or recurrent disease. In this study, we sought to investigate whether advanced imaging modalities may play a role in predicting the biological nature of meningiomas and/or serving as a predictive imaging biomarker to guide and optimize clinical management. To that end, we explored the relationship between DCE-perfusion parameters and [^68^Ga]-DOTATATE PET/MRI standardized uptake value (*SUV*) to determine whether a relationship may exist between vascularity, as represented by DCE perfusion parameters, and SSTR2 expression, as represented by [^68^Ga]-DOTATATE *SUV*.

## Materials and Methods

### Study Design

In this institutional review board-approved prospective study, patients with clinically suspected or histologically proven meningioma were enrolled as part of our active clinical trial and underwent [^68^Ga]-DOTATATE PET/MRI with DCE perfusion between August 2018 and April 2021. Patient exclusion criteria included contraindications to gadolinium-based contrast agents, a history of an allergic reaction to [^68^Ga]-DOTATATE, and pregnancy. Patients with histologically proven and/or with one histologically proven and additional suspected meningioma(s) on the basis of conventional MRI and measuring ≥1 cm in size in at least one dimension were included. In patients with multiple meningiomas, WHO grade of the histologically proven meningioma was assumed for all meningiomas present (18). [^68^Ga]-DOTATATE has been widely used in the diagnosis, staging, and treatment management of neuroendocrine tumors with a favorable safety profile (19), including lack of significant toxicity, lower radiation exposure, and improved accuracy compared to indium-111-pentetreotide (19).

### Imaging

All patients underwent gadolinium-enhanced MR imaging of the brain on a 3-Tesla clinical scanner (Biograph mMR, Siemens Healthcare, Erlangen, Germany), which included 3‐dimensional T1 SPACE (TR/TE, 600–700 ms/11–19 ms, 120°C flip, 1 mm slice thickness), and 3‐dimensional T2 FLAIR (TR/TE, 6,300–8,500 ms/394–446 ms, 120°C flip, 1 mm slice thickness). T1‐weighted DCE perfusion MRI was performed and available for all patients (TR = 4 ms; TE = 1–2 ms; flip angle, 13°C; slice thickness, 3 mm; 44 slices to cover the entire lesion volume; 24 phases with 4 phases before and 20 phases after intravenous bolus administration of 0.1 ml/kg gadobutrol).

PET acquisition was performed in dynamic 3D list mode for a total of 60 min starting simultaneously with [^68^Ga]-DOTATATE injection and concurrent with the above-described MR sequences. Absolute maximum SUV was extracted for each lesion, and SUV of the superior sagittal sinus (SSS), serving as background blood pool for normalization purposes based on previously published methodology (11).

### DCE Perfusion Analysis

**Figure 1:**
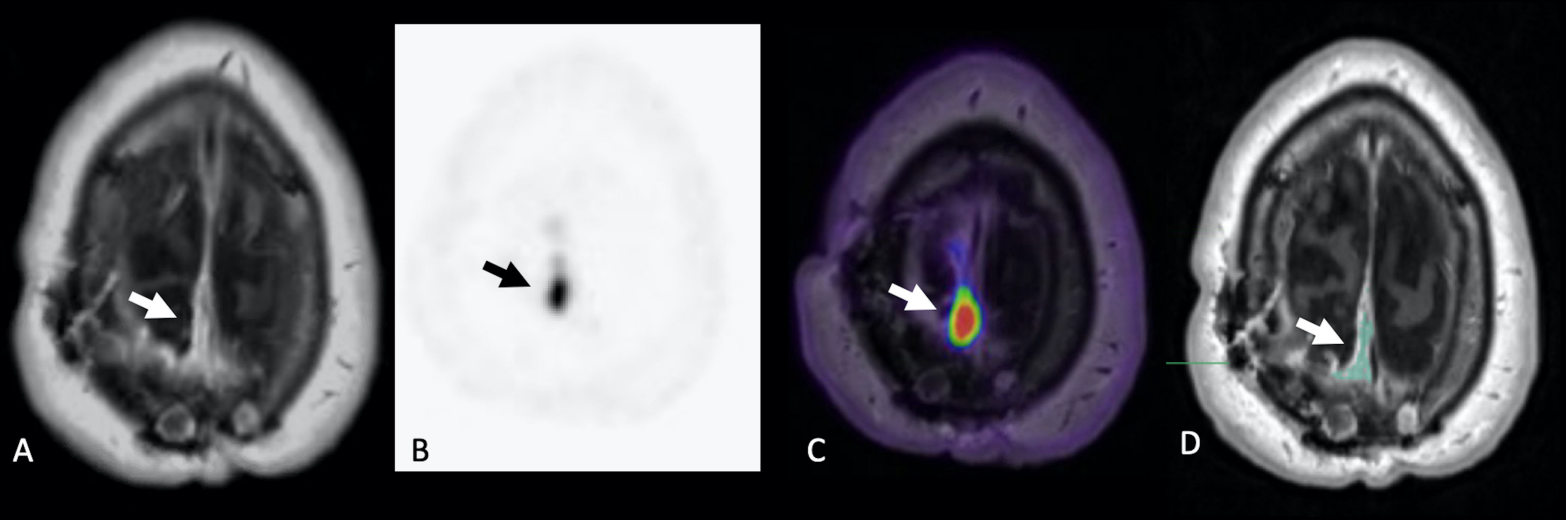
Example of VOI segmentation for DCE analysis in a 56-year-old man with WHO-2 right parietal meningioma post prior surgical resection and RT. Gadolinium-enhanced axial T1-weighted MRI **(A)**, static 68-Gallium-DOTATATE PET image **(B)**, and fused axial PET/MR **(C)** demonstrate extra-axial homogeneously enhancing soft tissue with corresponding 68-Gallium-DOTATATE avidity (arrows), compatible with residual/recurrent meningioma. Gadolinium-enhanced axial T1-weighted MRI with volume of interest **(D)** is shown, corresponding to region of suspected residual/recurrent meningioma.

**Figure 2:**
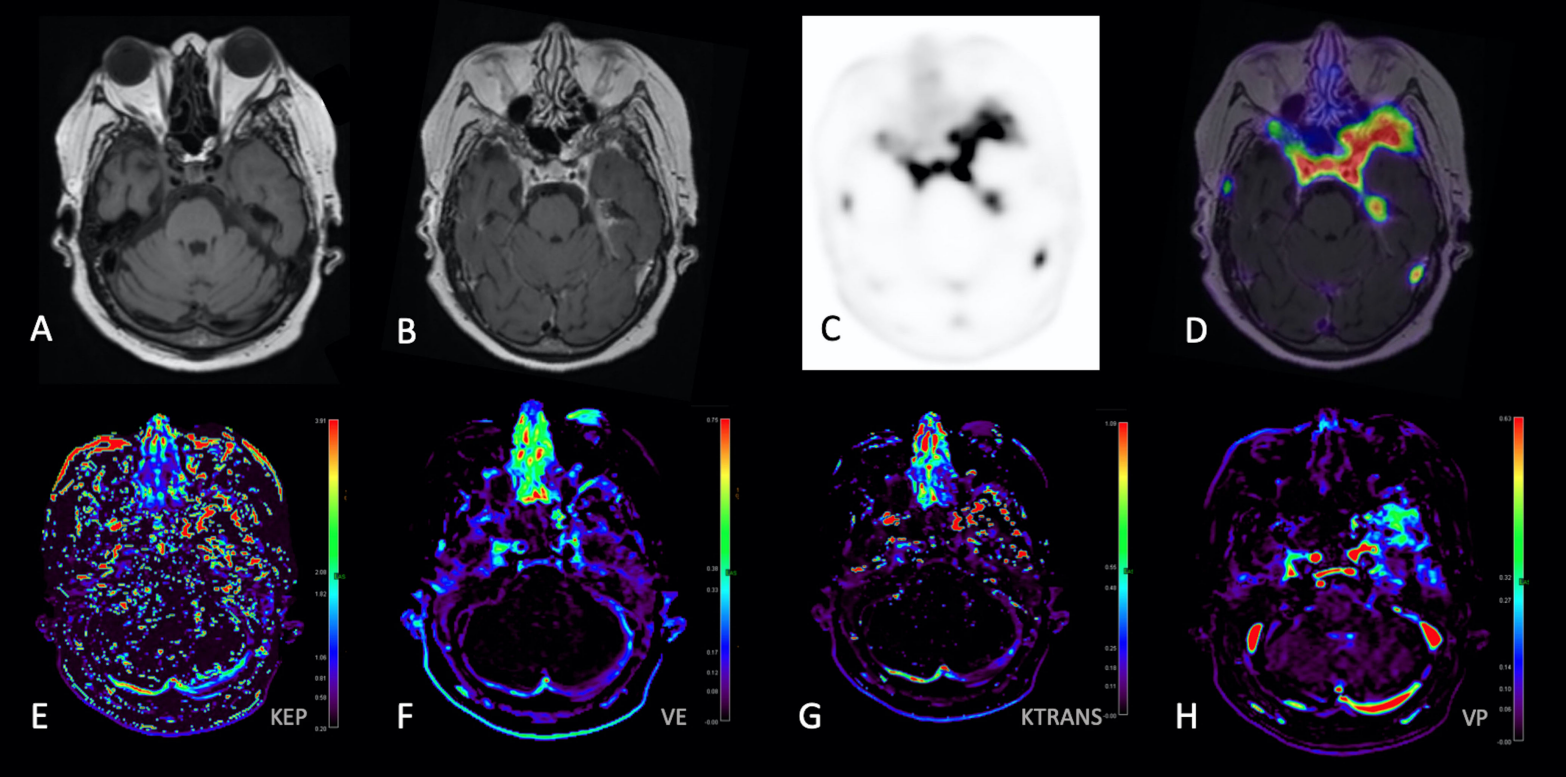
68-Gallium-DOTATATE PET and DCE Perfusion MRI images in a 68-year-old woman with WHO-1 left anterior temporal convexity meningioma post prior surgical resection and RT. Axial T1-weighted **(A)**, gadolinium-enhanced axial T1-weighted **(B)**, static 68-Gallium-DOTATATE PET image **(C)**, axial PET/MR fusion image **(D)**, Kep parametric map **(E)**, Ve parametric map **(F)**, Ktrans parametric map **(G)**, and Vp parametric map demonstrates extra-axial homogeneously enhancing soft tissue with corresponding 68-Gallium-DOTATATE avidity [**(D)**, with SUV measured along the left anterior temporal convexity] and abnormal perfusion parametric maps **(E–H)**, corresponding to region of suspected residual/recurrent meningioma.

DCE perfusion analysis was performed using Olea Sphere Medical 3.0-SP22 software (Olea Medical, La Ciotat, France). For each imaging study, a single investigator (MR) created a volume of interest (VOI) encompassing the meningioma utilizing the postcontrast T1-weighted sequence with intermittent guidance of the fused MRI/PET scan (e.g., in cases where conventional MRI was difficult to discern residual meningioma from adjacent dura), as shown as in **Figure 1**. The following Extended Tofts model DCE perfusion parameters were derived and analyzed from each VOI: flux rate constant (*Kep*), transfer constant (*Ktrans*), volume fraction of the extravascular extracellular space (*Ve*) in the tissue, and volume fraction of plasma in the tissue (*Vp*) (**Figure 2**). Briefly, these parameters are based upon a two-compartment model and the principle that intravenously injected contrast agent leaks from the intravascular space (IVS; compartment 1) into the EVS (compartment 2), and whether or not the tracer is freely diffusible. The rate of contrast exchange between these two compartments are described using transfer rate constants, including *Ktrans* (forward volume transfer constant), *Kep* (flux rate constant between EES and IVS), *Ve* (extracellular extravascular volume fraction whereby Ve = Ktrans/Kep), and *Vp* (plasma per unit volume of tissue).

In a randomly selected sample of eight meningiomas, volumetric segmentation and DCE perfusion analysis was repeated by a second investigator (SG).

### Statistical Analysis

Statistical analysis was performed using R version 4.0.5 (R Foundation for Statistical Computing, Vienna, Austria). Pearson correlation coefficient was calculated to identify whether a statistically significant correlation existed between DCE permeability parameters and [^68^Ga]-DOTATATE SUV. Intraclass correlation coefficient (ICC) was determined to assess interrater reliability between the two investigators.

## Results

Study cohort demographics are described in **Table 1**. This prospective study included 36 patients, with mean age of 53.6 years (range, 21–83; standard deviation, 14.8 years), of whom 61% (22 of 36) were female. A total of 60 meningiomas (20 WHO-1, 27 WHO-2, and 13 WHO-3) with average tumor volume of 2.3 cc (range, 0.04–26.11 cc; standard deviation, 4.25 cc) were included in this analysis. Of the 60 meningiomas included in our study, 30% (18/60) were located in the skull base. Of the 60 lesions in the cohort, 43 lesions were considered pathology-proven (72%).

**Table 1:** Clinical characteristics of the patient population.

N Patients	36
Age	53.6 (21–83; STD: 14.8)
Sex	61% F (22/36)
N meningiomas identified on PET	60
Tumor volume	2.3 (.04–26.11; STD: 4.25)
N meningiomas per patient	1 meningioma: 63.9% (24/36)
	33% (8/24) WHO Grade 1
	42% (10/24) WHO Grade 2
	25% (6/24) WHO Grade 3
	2-3 meningiomas: 25% (9/36)
	56% (5/9) WHO Grade 1
	33% (3/9) WHO Grade 2
	11% (1/9) WHO Grade 3
	≥4 meningiomas: 11.1% (3/36)
	0% (0/3) WHO Grade 1
	67% (2/3) WHO Grade 2
	33% (1/3) WHO Grade 3
	Median:1 meningioma per patient
WHO grade	0% (0/36) WHO grade unknown
	36% (13/36) WHO grade 1
	42% (15/36) WHO grade 2
	22% (8/36) WHO grade 3
Surgical history	94% (34/36)
Time from surgery to PET	26.5 months (1.4–118 months)
Prior radiation history	50% (18/36)
Prior radiation type	56% (10/18) SRS
	17% (3/18) gamma knife
	22% (4/18) proton
	6% (1/18) IMRT
Time from prior radiation to PET	31.6 months (0.26–205 months)
Radiation dose	41.5 Gy (5–123)

*Kep* demonstrated a strong significant positive correlation with [^68^Ga]-DOTATATE SUV (r = 0.84, p < 0.0001), which remained robust when normalized to background blood pool SSS SUV (SUVR_SSS_) (r = 0.81, p < 0.0001). When stratifying by WHO Grade, this strong significant positive correlation only existed in WHO-2 (r = 0.91, p < 0.0001; SUVR_SSS_, r = 0.91, p < 0.0001) and WHO-3 (r = 0.92, p = 0.0029; SUVR_SSS_, r = 0.82, p = 0.023) but did not exist with WHO-1 (r = 0.26, p = 0.4; SUVR_SSS_, r = 0.22, p = 0.46).

*Ktrans* demonstrated a moderate significant positive correlation with [^68^Ga]-DOTATATE SUV (r = 0.39, p = 0.019), which did not remain statistically significant with SUVR_SSS_ (r = 0.28, p = 0.11), and did not remain statistically significant when stratifying by WHO Grade.

When analyzing separately only lesions located in the skull base [30% (18/60)], there remained a strong positive significant correlation between *Kep* and SUV (r = 0.91, p < 0.0001) and a moderate positive significant correlation between Ktrans and SUV (r = 0.50, p = 0.04).

When analyzing separately only the pathology proven lesions [72% (43/60)], there remained a strong positive significant correlation between *Kep* and SUV (r = 0.72, p < 0.0001) and a moderate positive significant correlation between Ktrans and SUV (r = 0.49, p = 0.0009).

**Figure 3:**
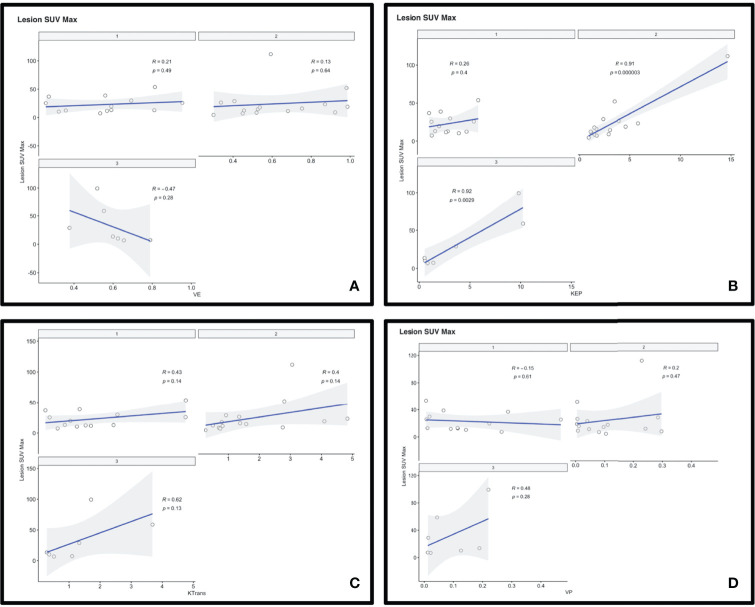
Scatterplots depicting the correlation between 68-Gallium-DOTATATE PET SUV and DCE perfusion parameters *Ve* **(A)**, *Kep* **(B)**, *Ktrans* **(C)**, and *Vp* **(D)**.

No other statistically significant correlation existed between [^68^Ga]-DOTATATE SUV and *Vp*, *Ve*, and *Ktrans*. All correlations are reported in **Tables 2**, **3** and **Figure 3**.

**Table 2:** SUV and DCE parameters.

SUV Lesion	25.06 (4.2–111.8; STD: 21.39)
SUV SSS	1.47 (0.6–2.5; STD: 0.5)
SUVR_SSS_ (SUV lesion/SSS)	18.55 (1.2–136.1; STD: 21.9)
*Kep*	3.18 (0.40–16.33; STD: 3.16)
*Ktrans*	1.68 (0.21–7.85; STD: 1.60)
*Vp*	0.10 (.0009–0.47; STD: 0.11)
*Ve*	0.56 (.091–0.99; STD: 0.21)

**Table 3:** SUV Correlations.

DCE Parameter to SUV		R	p-value
*Kep*	WHO Grade 1	0.26	0.4
	WHO Grade 2	0.91	< 0.001
	WHO Grade 3	0.92	< 0.01
*Ktrans*	WHO Grade 1	0.43	0.14
	WHO Grade 2	0.40	0.14
	WHO Grade 3	0.62	0.13
*Vp*	WHO Grade 1	-0.15	0.61
	WHO Grade 2	0.2	0.47
	WHO Grade 3	0.48	0.28
*Ve*	WHO Grade 1	0.21	0.49
	WHO Grade 2	0.13	0.64
	WHO Grade 3	-0.47	0.28
**DCE Parameters to SUVR_SSS_**			
*Kep*	WHO Grade 1	0.22	0.46
	WHO Grade 2	0.91	< 0.001
	WHO Grade 3	0.82	0.02
*Ktrans*	WHO Grade 1	0.23	0.45
	WHO Grade 2	0.4	0.14
	WHO Grade 3	0.46	0.3
*Vp*	WHO Grade 1	.0012	1
	WHO Grade 2	0.21	0.44
	WHO Grade 3	0.55	0.2
*Ve*	WHO Grade 1	-0.028	0.93
	WHO Grade 2	0.17	0.55
	WHO Grade 3	-0.32	0.48

The intraclass correlation coefficients (ICCs) for perfusion parameters *Kep*, *Vp*, and *Ve* were excellent: 0.998, 0.990 and 0.992, respectively. The ICC for *Ktrans* was unable to be calculated, and, upon directed review, an error was detected in the sampling of one meningioma resulting in an outlier data point for that measurement by one of the two readers. Upon exclusion of that data point, the ICC for *Ktrans* was also found to be excellent: 0.967. There was only a moderate reliability of assessed tumor volume, 0.742.

## Discussion

Dynamic contrast-enhanced MRI utilizes the acquisition of multiple serial images before, during, and after a bolus of low-molecular weight gadolinium contrast media, which allows for the determination of measurements of enhancement as a function of time (12, 20). Intravenously injected contrast material passes from the arteries to the tissue microvasculature and extravasates within seconds to the extravascular extracellular space (EES), or leakage space, resulting in shortening of the local relaxation time. DCE-MRI subsequently uses this T1 shortening (i.e., high signal or enhancement on T1-weighted sequence) to detect areas of BBB disruption. The ability to assess contrast agent extravasation, or vessel leakiness, is complex and relies on several factors, most notably blood flow. Therefore, the signal measured with DCE-MRI, particularly when using a sufficiently long acquisition time, reflects both perfusion and permeability and DCE-MRI can be impacted by alterations in vascular permeability, blood flow, and EES. Perfusion imaging may alternatively be performed with DSC-MRI, relying on T2*-weighted gradient-echo echo-planar imaging, which has several theoretical differences and a number of advantages, including faster acquisition, higher temporal resolution, and the ability to determine relative cerebral blood volume (rCBV), a widely used variable to assess tumor vascularity and grade (21).

A number of complex pharmacokinetic models have been proposed including Tofts et al. (22), Brix et al. (23), and Larsson et al. (24), many of which rely on a two-compartment model and the principle that intravenously injected contrast agent leaks from the intravascular space (IVS; compartment 1) into the EVS (compartment 2), and whether or not the tracer is freely diffusible. Most pharmacokinetic models determine the rate of contrast exchange between these two compartments using transfer rate constants, including *Ktrans* (forward volume transfer constant), *Kep* (flux rate constant between EES and IVS), *Ve* (extracellular extravascular volume fraction whereby Ve = Ktrans/Kep), and *Vp* (plasma per unit volume of tissue).

An initial model of BBB permeability was developed by Tofts et al., ignoring the contribution of plasma to total tissue concentration and consequently only applicable in normal brain tissue with an intact BBB. A subsequent model, the Extended Tofts model, incorporates the vascular contribution to signal intensity and is more commonly used in tumor applications, including in this analysis (25). While quantitative DCE-MRI measurements and parametric maps are increasingly used for diagnostic purposes, it is critical to understand the complexity of these measurements and the numerous variables that affect their results, including the arterial input function (AIF; i.e., measured concentration in an artery) and physiological factors (e.g., changes in cardiac output), for which determination of consistent and accurate data, both in the clinical and research settings, may be a challenge. Of note, the data presented in this analysis demonstrated an excellent ICC across the assessed parameters. The assessed tumor volume demonstrated only moderate reliability, suggesting robust DCE analysis results even with varying sampled volume. Therefore, the consistency of data obtained by two independent observers supports the accuracy of the results presented.

Prior immunohistochemical analyses investigating the relationship of vascularity in meningiomas identified a significant upregulation of VEGF-A in WHO Grade III as compared to WHO Grade II tumors (26). In this radiological analysis, the only perfusion parameter to demonstrate statistical significance with [^68^Ga]-DOTATATE SUV was *Kep*, a parameter infrequently used in clinical practice but often described in the literature. Awasthi et al. investigated whether an association may exist in glioblastoma (GBM) between DCE-MRI parameters and tissue matrix metalloproteinase 9 (MMP-9) expression, with MMPs known to be responsible for targeting the extracellular matrix and contributing to BBB permeability and angiogenesis/neovascularization of glial tumors. In their study, they determined that MMP-9 expression was best estimated by *Kep*, of all perfusion parameters, and demonstrated an association with survival, suggesting *Kep* as a potential imaging biomarker of GBM progression and its prognostication (27).

It is important to note, however, that GBM are intra-axial in location while meningiomas are extra-axial in location, raising the question as to whether tumor origin impacts the interpretation of DCE-MRI findings. Physiological extreme vessel leakiness is observed with both tumor types—in GBM due to BBB destruction of preexisting vessels and faulty BBB in angiogenic tumoral vessels, while in meningiomas due to their highly vascular nature and inherent absence of a BBB given their extra-axial location (21). Cha et al. compared DCE-MRI and DSC-MRI microvascular permeability measurement *Ktrans* in gliomas and meningiomas, observing that *Ktrans* could distinguish between higher- and lower-grade gliomas, although correlated poorly in meningiomas (21). However, Chidambaram et al. investigated DCE-MRI in meningiomas treated with resection and adjuvant radiosurgery, revealing a moderately positive correlation with *Ktrans* and time to progression, approaching but not reaching statistical significance, which supports a role of DCE-MRI as a biomarker in meningioma diagnosis, treatment planning, and predicting clinical outcomes (13).

A study evaluating DSC-MRI characteristics of meningiomas compared to dural-based metastases by Lui et al. identified relative wash-in time, a metric describing the wash-in phase of perfusion, to be lower in metastases as compared to meningiomas. This study also investigated the use of rCBV, a metric that is frequently used to assess tumor vascularity and grade, in the distinction of meningioma from dural-based metastases. However, in distinction to other reports describing the utility of rCBV for extra-axial lesions, the use of rCBV was found to be limited within this patient cohort (28). While many of the aforementioned studies utilized DSC-MRI and did not specifically investigate the perfusion parameter *Kep*, the overarching theme among these studies is in support of the potential role and added value of perfusion imaging for the assessment of meningiomas.

The significance of *Kep* with respect to other tumor types has been previously described in the literature. For example, *Kep* has been shown to negatively correlate with histological vessel maturity in breast cancer osseous metastases (29), positively correlate with Ki67 and p53- and triple negative status in breast cancers (30), positively correlate with invasive ductal carcinoma tumor size (31), and positively correlate with microvessel density (32) and PTEN expression in prostate cancer (33). *Kep* has also been shown to effectively differentiate between benign and malignant soft tissue tumors (34) and demonstrate a significant positive correlation with serum angiogenesis-related biomarkers and advanced tumor stage in patients with non-small cell lung cancer (35). The relationship between *Kep* and SSTR2A expression in higher grade meningiomas may be of significance in the treatment planning and response assessment of meningiomas. To this end, bevacizumab, a monoclonal antibody targeting vascular endothelial growth factor (VEGF), has been used in meningioma off label with reports of improved overall PFS (36). The mechanism of action may be related to decrease in vascularity and associated decrease in SSTR2A expression, which may serve as a clinical response biomarker. The identified correlation between DCE-MRI perfusion parameter *Kep* and [^68^Ga]-DOTATATE *SUV* in higher grade meningiomas suggests underlying biological differences in the relationship between tumor vascularity and SSTR2 expression, perhaps related to biomarkers of angiogenesis, such as VEGF and microvessel density. This may be of importance given ongoing efforts to apply peptide receptor radionuclide therapy (PPRT) with ^177^Lutetium[^177^Lu]-DOTATATE in meningioma, which has been reported to have modest effects in small pilot cohorts and individual cases (2, 37, 38). To that end, PRRT with [^177^Lu]-DOTATATE in gastrointestinal neuroendocrine tumors metastatic to the liver was recently shown to have improved dosimetry with intra-arterial interventional radiology-guided administration compared to systemic intravenous administration (39). Conceivably, the correlation between perfusion metrics and [68Ga]-DOTATATE PET SUV may thus play a role when determining patients for clinical trials incorporating PRRT who are most likely to benefit. The relationship between *Kep* and SUV may also be clinically relevant to other therapeutic options that specifically address tumor vascularity.

A limitation of this study is the relatively small sample size, with 36 patients with 60 meningiomas. Additionally, in patients with multiple meningiomas and WHO grade documented for one meningioma, the assumption was made for the additional unresected lesions to have the same WHO grade, as previously published (18). While this approach has previously been validated, there remains the theoretical possibility for heterogeneity of WHO grades in patients with multiple meningiomas. While this study only included meningiomas measuring ≥1 cm in at least one dimension, some of the sampled tumor volumes were relatively low, which raises the possibility of partial volume averaging effects in these smaller sampled volumes. Furthermore, 50% (18/36) of meningiomas within this cohort had received prior RT, potentially serving as a confounder for our data correlating vascularity with [^68^Ga]-DOTATATE avidity. Future work assessing this correlation in treatment-naive meningiomas is warranted. Additionally, future immunohistochemical investigation utilizing SSTR2 stains and those investigating vascularity on resected tumor samples can assist in supporting the results of this study. Finally, future work evaluating the cost effectiveness of utilizing [^68^Ga]-DOTATATE PET in meningioma management will be important, as this approach may ultimately reduce costs related to decreased complications from RT and has the potential to improve progression-free survival by improving targeted radiation dose delivery in patients with small volume residual disease (7).

## Conclusions

In this study, we found a strong, significant correlation between the DCE-MRI perfusion parameter *Kep* and [^68^Ga]-DOTATATE *SUV* in WHO Grade 2/3 meningiomas, which suggests biological differences in the relationship between tumor vascularity and SSTR2 expression in higher-grade meningiomas. Our findings may have pathophysiological implications for clinical management of patients with meningiomas. Future work to understand the potential prognostic role of combined DOTATATE PET and DCE MRI in meningioma treatment planning and response assessment is warranted.

## Data Availability Statement

The original contributions presented in the study are included in the article/supplementary material. Further inquiries can be directed to the corresponding author.

## Ethics Statement

The studies involving human participants were reviewed and approved by New York-Presbyterian Hospital, Weill Cornell Medicine. The patients/participants provided their written informed consent to participate in this study. Written informed consent was obtained from the individual(s) for the publication of any potentially identifiable images or data included in this article.

## Author Contributions

All authors provided contributions to study conception and design, acquisition of data or analysis and interpretation of data, drafting the article or revising it critically for important intellectual content, and final approval for publication. Most important contributions of each author are as follows: conceptualization, JI and MR; data curation, MR, SK, SG, CT, EL, WF, RM, TS, RR, NK, SP, and JK; formal analysis, MR, SK, SG, CT, and JI; resources, RM, TS, RR, SP, JO, JK, and JI; writing—original draft, MR and JI; writing—review and editing, MR, EL, RM, BL, TS, RR, NK, SP, JO, JK, and JI.

## Funding

This study was designed, conducted, analyzed, and reported entirely by the authors. This paper presents independent research funded by the 2019-2020 RSNA Radiology Resident Research Grant (#RR1962 PI: MR), the 2021-2022 RSNA Medical Student Research Grant (PI: SK), Weill Cornell Medical College (PI: JI) and Novartis Pharmaceuticals (Investigator-Initiated Trial, PI: JI). Novartis Pharmaceuticals was not involved in the study design, collection, analysis, interpretation of data, the writing of this article or the decision to submit it for publication.

## Conflict of Interest

The authors declare that the research was conducted in the absence of any commercial or financial relationships that could be construed as a potential conflict of interest.

## Publisher’s Note

All claims expressed in this article are solely those of the authors and do not necessarily represent those of their affiliated organizations, or those of the publisher, the editors and the reviewers. Any product that may be evaluated in this article, or claim that may be made by its manufacturer, is not guaranteed or endorsed by the publisher.

## References

[1] Lum MA Martin AJ Alexander MD McCoy DB Cooke DL Lillaney P. Intra-Arterial MR Perfusion Imaging of Meningiomas: Comparison to Digital Subtraction Angiography and Intravenous Mr Perfusion Imaging. PloS One (2016) 11:1–13. doi: 10.1371/journal.pone.0163554 PMC5089755 27802268

[2] Cordova C Kurz SC. Advances in Molecular Classification and Therapeutic Opportunities in Meningiomas. Curr Oncol Rep (2020) 22:1–10. doi: 10.1007/s11912-020-00937-4 32617743

[3] Louis DN Perry A Wesseling P Brat DJ Cree IA Figarella-Branger D. The 2021 WHO Classification of Tumors of the Central Nervous System: A Summary. Neuro Oncol (2021) 23:1231–51. doi: 10.1093/neuonc/noab106 PMC8328013 34185076

[4] Ansari SF Shah KJ Hassaneen W Cohen-Gadol AA. Vascularity of Meningiomas. Handb Clin Neurol (2020) 169:153–65. doi: 10.1016/B978-0-12-804280-9.00010-X 32553286

[5] Rogers L Barani I Chamberlain M Kaley TJ McDermott M Raizer J. Meningiomas: Knowledge Base, Treatment Outcomes, and Uncertainties. A RANO Review. J Neurosurg (2015) 122:4–23. doi: 10.3171/2014.7.JNS131644 25343186 PMC5062955

[6] Gallagher MJ Jenkinson MD Brodbelt AR Mills SJ Chavredakis E. WHO Grade 1 Meningioma Recurrence: Are Location and Simpson Grade Still Relevant? Clin Neurol Neurosurg (2016) 141:117–21. doi: 10.1016/j.clineuro.2016.01.006 26780494

[7] Mahase SS Roth O’Brien DAR No D Roytman M Skafida ME Lin E. [68ga]-DOTATATE PET/MRI as an Adjunct Imaging Modality for Radiation Treatment Planning of Meningiomas. Neuro-Oncol Adv (2021) 3:1–9. doi: 10.1093/noajnl/vdab012 PMC7954102 33738446

[8] Roytman M Pisapia DJ Liechty B Lin E Skafida M Magge RS. Somatostatin Receptor-2 Negative Meningioma: Pathologic Correlation and Imaging Implications. Clin Imaging (2020) 66:18–22. doi: 10.1016/j.clinimag.2020.04.026 32442855

[9] Ivanidze J Roytman M Sasson A Skafida M Fahey TJ III Osborne JR. Molecular Imaging and Therapy of Somatostatin Receptor Positive Tumors. Clin Imaging (2019) 56:146–54. doi: 10.1016/j.clinimag.2019.04.006 31121520

[10] Hofman MS Eddie Lau WF Hicks RJ. Somatostatin Receptor Imaging With 68 Ga DOTATATE PET/CT: Clini- Cal Utility, Normal Patterns, Pearls, and Pitfalls in Interpretation. Radioraphics (2016) 35:500–16. doi: 10.1148/rg.352140164 25763733

[11] Ivanidze J Roytman M Lin E Magge RS Pisapia DJ Liechty B. Gallium-68 DOTATATE PET in the Evaluation of Intracranial Meningiomas. J Neuroimaging (2019) 0:1–7. doi: 10.1111/jon.12632 31107591

[12] Heye AK Culling RD Valdés Hernández MC Thrippleton MJ Wardlaw JM. Assessment of Blood-Brain Barrier Disruption Using Dynamic Contrast-Enhanced MRI. A Systematic Review. NeuroImage Clin (2014) 6:262–74. doi: 10.1016/j.nicl.2014.09.002 PMC4215461 25379439

[13] Chidambaram S Pannullo SC Roytman M Pisapia DJ Liechty B Magge RS. Dynamic Contrast-Enhanced Magnetic Resonance Imaging Perfusion Characteristics in Meningiomas Treated With Resection and Adjuvant Radiosurgery. Neurosurg Focus (2019) 46:1–6. doi: 10.3171/2019.3.FOCUS1954 31153141

[14] Tamrazi B Shiroishi MS Liu CSJ. Advanced Imaging of Intracranial Meningiomas. Neurosurg Clin N Am (2016) 27:137–43. doi: 10.1016/j.nec.2015.11.004 PMC4936906 27012378

[15] Zhang H Rödiger LA Shen T Miao J Oudkerk M. Preoperative Subtyping of Meningiomas by Perfusion MR Imaging. Neuroradiology (2008) 50:835–40. doi: 10.1007/s00234-008-0417-3 18542938

[16] Yang S Law M Zagzag D Wu HH Cha S Golfinos JG. Dynamic Contrast-Enhanced Perfusion MR Imaging Measurements of Endothelial Permeability: Differentiation Between Atypical and Typical Meningiomas. Am J Neuroradiol (2003) 24:1554–9. PMC7974003 13679270

[17] Sacco S Ballati F Gaetani C Lomoro P Farina LM Bacila A. Multi-Parametric Qualitative and Quantitative MRI Assessment as Predictor of Histological Grading in Previously Treated Meningiomas. Neuroradiology (2020) 62:1441–9. doi: 10.1007/s00234-020-02476-y 32583368

[18] Sommerauer M Burkhardt J Frontzek K Rushing E Buck A Krayenbuehl N. 68Gallium-DOTATATE PET in Meningioma: A Reliable Predictor of Tumor Growth Rate? Neuro Oncol (2016) 18:1021–7. doi: 10.1093/neuonc/now001 PMC4896546 26865086

[19] Deppen SA Liu E Blume JD Clanton J Shi C Jones-Jackson LB. Safety and Efficacy of 68Ga-DOTATATE PET/CT for Diagnosis, Staging, and Treatment Management of Neuroendocrine Tumors. J Nucl Med (2016) 57:708–14. doi: 10.2967/jnumed.115.163865 PMC5362940 26769865

[20] Verma S Turkbey B Muradyan N Rajesh A Cornud F Haider MA. Overview of Dynamic Contrast-Enhanced MRI in Prostate Cancer Diagnosis and Management. Am J Roentgenol (2012) 198:1277–88. doi: 10.2214/AJR.12.8510 PMC6309691 22623539

[21] Cha S Yang L Johnson G Lai A Chen M-H Tihan T. Comparison of Microvascular Permeability Measurements, Ktrans, Determined With Conventional Steady-State T1-Weighted and First-Pass T2*-Weighted MR Imaging Methods in Gliomas and Meningiomas. Am J Neuroradiol (2006) 27:409–17. PMC8148770 16484420

[22] Tofts PS Brix G Buckley DL Evelhoch JL Henderson E Knopp MV. Estimating Kinetic Parameters From Dynamic Contrast-Enhanced T1-Weighted MRI of a Diffusable Tracer. J Magn Reson Imaging (1999) 10:223–32. doi: 10.1002/(SICI)1522-2586(199909)10:3<223::AID-JMRI2>3.0.CO;2-S 10508281

[23] Brix G Semmler P Rudiger S Lothar R Layer G Lorenz WJ. Pharmacokinetic Parameters in Cns Gd-Dtpa Enhanced Mr Imaging. J Comput Assist Tomogr (1991) 15:621–8. doi: 10.1097/00004728-199107000-00018 2061479

[24] Larsson HBW Stubgaard M Frederiksen JL Jensen M Henriksen O Paulson OB. Quantitation of Blood-Brain Barrier Defect by Magnetic Resonance Imaging and Gadolinium-DTPA in Patients With Multiple Sclerosis and Brain Tumors. Magn Reson Med (1990) 16:117–31. doi: 10.1002/mrm.1910160111 2255233

[25] Sourbron SP Buckley DL. On the Scope and Interpretation of the Tofts Models for DCE-MRI. Magn Reson Med (2011) 66:735–45. doi: 10.1002/mrm.22861 21384424

[26] Bernatz S Monden D Gessler F Radic T Hattingen E Senft C. Influence of VEGF-A, VEGFR-1-3, and Neuropilin 1-2 on Progression-Free: And Overall Survival in WHO Grade II and III Meningioma Patients. J Mol Histol (2021) 52:233–43. doi: 10.1007/s10735-020-09940-2 PMC8012320 33528717

[27] Awasthi R Pandey CM Sahoo P Behari S Kumar V Kumar S. Dynamic Contrast-Enhanced Magnetic Resonance Imaging-Derived K Ep as a Potential Biomarker of Matrix Metalloproteinase 9 Expression in Patients With Glioblastoma Multiforme: A Pilot Study. J Comput Assist Tomogr (2012) 36:125–30. doi: 10.1097/RCT.0b013e31823f6c59 22261782

[28] Lui YW Malhotra A Farinhas JM Dasari SB Weidenheim K Freeman K. Dynamic Perfusion MRI Characteristics of Dural Metastases and Meningiomas: A Pilot Study Characterizing the First-Pass Wash-in Phase Beyond Relative Cerebral Blood Volume. Am J Roentgenol (2011) 196:886–90. doi: 10.2214/AJR.10.5309 21427341

[29] Merz M Seyler L Bretschi M Semmler W Bauerle T. Diffusion-Weighted Imaging and Dynamic Contrast-Enhanced MRI of Experimental Breast Cancer Bone Metastases - A Correlation Study With Histology. Eur J Radiol (2015) 84:623–30. doi: 10.1016/j.ejrad.2015.01.002 25641009

[30] Kang SR Kim HW Kim HS. Evaluating the Relationship Between Dynamic Contrast-Enhanced MRI (DCE-MRI) Parameters and Pathological Characteristics in Breast Cancer. J Magn Reson Imaging (2020) 52:1360–73. doi: 10.1002/jmri.27241 32524658

[31] Liu F Wang M Li H. Role of Perfusion Parameters on DCE-MRI and ADC Values on DWMRI for Invasive Ductal Carcinoma at 3.0 Tesla. World J Surg Oncol (2018) 16:1–12. doi: 10.1186/s12957-018-1538-8 30577820 PMC6303963

[32] Schlemmer H-P Merkle J Grobholz R Jaeger T Michel MS Werner A. Can Pre-Operative Contrast-Enhanced Dynamic MR Imaging for Prostate Cancer Predict Microvessel Density in Prostatectomy Specimens? Eur Radiol (2004) 14:309–17. doi: 10.1007/s00330-003-2025-2 14531000

[33] McCann SM Jiang Y Fan X Wang J Antic T Prior F. Quantitative Multiparametric MRI Features and PTEN Expression of Peripheral Zone Prostate Cancer: A Pilot Study. Am J Roentgenol (2016) 206:559–65. doi: 10.2214/AJR.15.14967 26901012

[34] Choi YJ Lee IS Song YS Kim JI Choi K Song JW. Diagnostic Performance of Diffusion-Weighted (DWI) and Dynamic Contrast-Enhanced (DCE) MRI for the Differentiation of Benign From Malignant Soft-Tissue Tumors. J Magn Reson Imaging (2019) 50:798–809. doi: 10.1002/jmri.26607 30663160

[35] Huang YS Chen JLY Chen HM Yeh LH Shih JY Yen RF. Assessing Tumor Angiogenesis Using Dynamic Contrast-Enhanced Integrated Magnetic Resonance-Positron Emission Tomography in Patients With non-Small-Cell Lung Cancer. BMC Cancer (2021) 21:1–13. doi: 10.1186/s12885-021-08064-4 33794813 PMC8017855

[36] Scerrati A Mongardi L Visani J Lofrese G Cavallo MA Fiorentino A. The Controversial Role of Bevacizumab in the Treatment of Patients With Intracranial Meningioma: A Comprehensive Literature Review. Expert Rev Anticancer Ther (2020) 20:197–203. doi: 10.1080/14737140.2020.1736567 32116057

[37] Seystahl K Stoecklein V Schüller U Rushing E Nicolas G Schafer N. Somatostatin Receptor-Targeted Radionuclide Therapy for Progressive Meningioma: Benefit Linked to 68Ga-DOTATATE/-TOC Uptake. Neuro Oncol (2016) 18:1538–47. doi: 10.1093/neuonc/now060 PMC5063513 27106404

[38] Makis W McCann K McEwan AJB. Rhabdoid Papillary Meningioma Treated With 177Lu DOTATATE PRRT. Clin Nucl Med (2015) 40:237–40. doi: 10.1097/RLU.0000000000000669 25608146

[39] Thakral P Sen I Das SS Manda D CB V Malik D. Dosimetric Analyses of Intra-Arterial Versus Standard Intravenous Administration of 177Lu-DOTATATE in Patients of Well Differentiated Neuroendocrine Tumor With Liver-Dominant Metastatic Disease. Br J Radiol (2021) 94:1–11. doi: 10.1259/bjr.20210403 PMC9328058 34357794

